# 801. Adherence to 20 Minutes of Cool Running Water for Acute Burns in Emergency Care

**DOI:** 10.1093/jbcr/irag033.179

**Published:** 2026-04-06

**Authors:** Maleea D Holbert, Tina L Palmieri, John S Rose, Logen Howze, Nathan Kuppermann, Bronwyn R Griffin

**Affiliations:** Queensland Children’s Hospital, Brisbane, QLD; University of California Davis Health, Shriners Children’s - Northern California, Sacramento, CA; University of California Davis Health, Sacramento, CA; University of California Davis Health, Sacramento, CA; Children's National Hospital and George Washington School of Medicine and Health Sciences, Washington, DC; Griffith University, Brisbane, QLD

## Abstract

**Introduction:**

Twenty minutes of cool running water (20CRW) is the recommended, evidence-based first-line treatment for acute thermal burn injuries in Australia, New Zealand, the United Kingdom, and across Europe. When delivered within the first three hours of a burn, this first aid treatment reduces burn depth progression and lowers the need for skin grafting. We assessed the implementation of 20CRW to the first 200 burn patient encounters in the emergency department (ED) of a major burn referral hospital from April 2024 – August 2025.

**Methods:**

ED clinicians (comprised medical, nursing, and technician staff) completed online questionnaires for the first 200 burn patient encounters following the implementation of 20CRW as standard first aid management for acute thermal injuries. Questionnaires assessed whether 20CRW was applied, reasons for not providing it when eligible, and perceived barriers to consistent implementation. Eligibility for 20CRW was predefined, with exclusions including burns >30% total body surface area, Glasgow Coma Scale score < 15, airborne or contact precautions, cervical spine immobilization, lack of intravenous access, requirement for escharotomy, presentation >3 hours post-burn, hypothermia, inhalation injury, and burns involving the face. Free-text data were analyzed using inductive content analysis in NVivo.

**Results:**

During the first 200 burn patient encounters, 95% (n = 190) were eligible for 20CRW provision. Among eligible patients, 80.5% (n = 153) received 20CRW within three hours of injury. Of these, 62.6% (n = 119) had 20CRW administered in the ED, and 17.9% (n = 34) in the prehospital setting prior to transport. An additional 4.7% (n = 9) received partial cooling (e.g., 5 – 15 minutes of cool running water). Of the 14.7% of eligible burn patients (n = 28) who did not receive first aid cooling, three chief reasons were identified:

1. Assorted barriers to 20CRW administration (n = 17; 8.9%): barriers such as limited access to showers, time constraints, staffing shortages, and high patient volumes.

2. Patient-related barriers (n = 5; 2.6%): combativeness, physical restraints, intoxication, age-related challenges, severe pain, and emotional distress.

3. Clinician-directed decisions (n = 6; 3.2%): four cases (2.1%) where emergency physicians advised against 20CRW; and two cases (1.1%) where burn surgeons recommended immediate alternative interventions (e.g., debridement).

**Conclusions:**

The implementation of 20CRW as a first aid treatment was successfully integrated into the ED, with 80.5% of eligible burn patients receiving timely cooling within three hours of injury. Targeted strategies, combined with this evidence-based intervention, may drive higher adherence rates.

**Applicability of Research to Practice:**

These findings highlight opportunities to improve adherence to first aid guidelines through targeted interventions addressing resource limitations, clinical decision-making, and patient compliance.

**Funding for the study:**

This research received competitive grant funding, awarded to the Principal Investigator.

